# Biological interpretation of the sporadic sputum smear-positive-culture-negative outcome for patients with tuberculosis undertaking treatments

**DOI:** 10.3389/fpubh.2023.1064512

**Published:** 2023-02-10

**Authors:** Jingjing Luo, Xia Yu, Lingling Dong, Fengmin Huo, Yifeng Ma, Qian Liang, Yuanyuan Shang, Hairong Huang

**Affiliations:** ^1^Department of Laboratory Medicine, West China Second University Hospital, Sichuan University, Chengdu, China; ^2^Key Laboratory of Birth Defects and Related Diseases of Women and Children (Sichuan University), Ministry of Education, Chengdu, China; ^3^National Clinical Laboratory on Tuberculosis, Beijing Key Laboratory for Drug-Resistant Tuberculosis Research, Beijing Tuberculosis and Thoracic Tumor Institute, Beijing Chest Hospital, Capital Medical University, Beijing, China

**Keywords:** tuberculosis, smear, culture, smear-positive-culture-negative, dead bacilli

## Abstract

**Introduction:**

The objective of the study was to identify the causes of smear-positive-culture-negative (S+/C–) outcomes of patients with tuberculosis during the treatment course.

**Methods:**

A laboratory-based retrospective study was performed at the Beijing Chest Hospital in China. Within the study period, all patients with pulmonary tuberculosis (PTB) who undertook anti-TB treatments and yielded smear positive outcomes with simultaneous culture outcomes on sputa were considered. Patients were classified into three groups: (I) performed LJ medium culture only; (II) performed BACTEC MGIT960 liquid culture only; and (III) performed both LJ culture and MGIT960 culture. The S+/C– rates of each group were analyzed. The clinical medical records regarding patient category, follow-up bacteriologic examination data, and treatment response were investigated.

**Results:**

In total, 1,200 eligible patients were enrolled, and the overall S+/C– rate was 17.5% (210/1,200). Group I had obviously higher S+/C– rate (37%) than group II (18.5%) and group III (9.5%). When solid and liquid cultures were considered independently, the S+/C– outcome was observed more frequently in the solid culture group than in the liquid culture group (30.4%, 345/1,135 vs. 11.5%, 100/873; *p* < 0.001, χ^2^ = 102.64). Among the 102 S+/C– patients who had follow-up cultures performed, 35 (34.3%) had positive culture outcomes. Whereas among the 67 patients with follow-up information for more than 3 months but without supportive bacteriological evidence, 45 (67.2%, 45/67) had unfavorable prognosis (including relapse and unimproved conditions), and only 22 (32.8%, 22/67) patients had improved conditions. Compared with new cases, retreated cases produced S+/C– outcomes more frequently and had more chances to be cultivated bacilli successfully afterward.

**Conclusions:**

Among our patients, the sporadic smear positive and culture negative outcomes for sputa are more likely associated with the technical failures of culture than with dead bacilli, and this is especially noteworthy for LJ medium culture.

## 1. Introduction

In addition to symptomatic and radiographic improvement, the absence of acid-fast bacilli (AFB) from sputum smears and negative culture outcome are used to assess the response to therapy for patients with tuberculosis (TB) (1, 2). Direct sputum smear microscopy for AFB is frequently the mainstay technique for pulmonary tuberculosis (PTB) patient diagnosis in resource-limited settings, while culture on the solid or liquid medium is the recognized “gold standard” for TB diagnosis. It is well-known that smear microscopy has poor sensitivity, particularly in patients with limited pulmonary involvement or immunosuppression (3). In contrast, both solid and liquid media have higher sensitivities and are, therefore, considered the most sensitive methods for mycobacterial recovery (4, 5). However, smear-positive sputa sometimes remain negative on culture in clinical practice (6), which could be associated with the excretion of dead bacilli from the patients (7, 8). According to previous studies, 20%−32% of the patients receiving anti-TB therapy could yield smear-positive-culture-negative (S+/C–) outcomes sometimes during the treatment course, and non-viable mycobacteria was presumed as the main cause (9). However, the mentioned studies had never considered the suboptimal sensitivity or technical failure of culture, which could also cause the S+/C– phenomenon. Various factors can impact successful culturing: chemical reagents used for liquefying sputa and decontamination may kill the bacilli in the specimen, bacilli loss due to inappropriate centrifugation, inappropriate transportation and storage and delayed processing of the specimens, etc. (10–12).

Identifying whether or not the dead bacilli cause the S+/C outcome is a very serious question. As dead bacilli mean that the treatment regimen is effective and should be continued, whereas if the suboptimal sensitivity of culture is the reason, smear positive could mean ill treatment response, leading to regimen adjustment, as well as public health action, such as elongated isolation of the patient and an expanded contact investigation. In this study, we sought to test whether laboratory technical failure is an important cause of S+/C– outcomes, which can help clinicians to better interpret the outcomes and then take appropriate action.

## 2. Methods

### 2.1. Ethics statement

This study was approved by the Medical Ethics Committee of Beijing Chest Hospital. The written consent was waived due to the retrospective design of the study.

### 2.2. Study design

A laboratory-based retrospective study was performed in the Beijing Chest Hospital, a designated hospital for TB patient care in Beijing, China. All smear positive outcomes from sputa of patients with PTB, undertaking anti-TB treatments, were considered from January 2015 to July 2016. The eligibility criteria for patient recruitment in this study were as follows: (I) The sputum smear test was positive, (II) Lowenstein-Jensen (LJ) medium culture and/or BACTEC MGIT 960 liquid culture were performed with the same specimen that produced positive smear test, or with specimen collected in the same day with the smear testing, and (III) negative outcome was obtained from the culturing. For patients with multiple S+/C– outcomes during the study period, the analysis was based on the time point of the first S+/C– outcome. The clinical medical records regarding patient category, follow-up bacteriologic examination data, and treatment outcomes were investigated.

### 2.3. Classification of patients

Patients were classified into three groups based on the culturing methods used when recruited for this study: (I) performed LJ medium culture only; (II) performed BACTEC MGIT960 medium culture only; and (III) performed both LJ medium and MGIT960 medium culture.

### 2.4. Microscopic examination and culture

Each sputum sample should contain 5–10 ml of sputum. Direct smear, prepared with sputum, was stained using auramine and then examined by light-emitting diode (LED) microscopy (Carl Zeiss AG, PrimoStar, Germany). The solid culture with LJ medium was performed following the guidelines from the Chinese anti-TB Association (13) and the MGIT960 culture was conducted according to the manufacturer's protocol. Briefly, after liquefied and decontaminated by the hydroxide-sodium citrate-N-actyl-L-cysteine method, 0.5 ml of sputum sediment was inoculated onto an LJ slant and incubated at 37°C for up to 8 weeks in an incubator, and/or inoculated 0.5 ml into a 7-ml MGIT tube and incubated in the BACTEC MGIT 960 system (BD Diagnostic Systems, Sparks, MD, USA). Positive cultures of MGIT960 were confirmed by examining Ziehl-Neelsen (ZN)-stained smears for AFB.

### 2.5. Statistical analysis

The statistical analysis was carried out using the χ^2^ test, with a *p*-value of <0.05 indicating significance, using SPSS version 13.0 software.

## 3. Results

### 3.1. The characteristics of the included smear+/culture–patients

**Figure 1:**
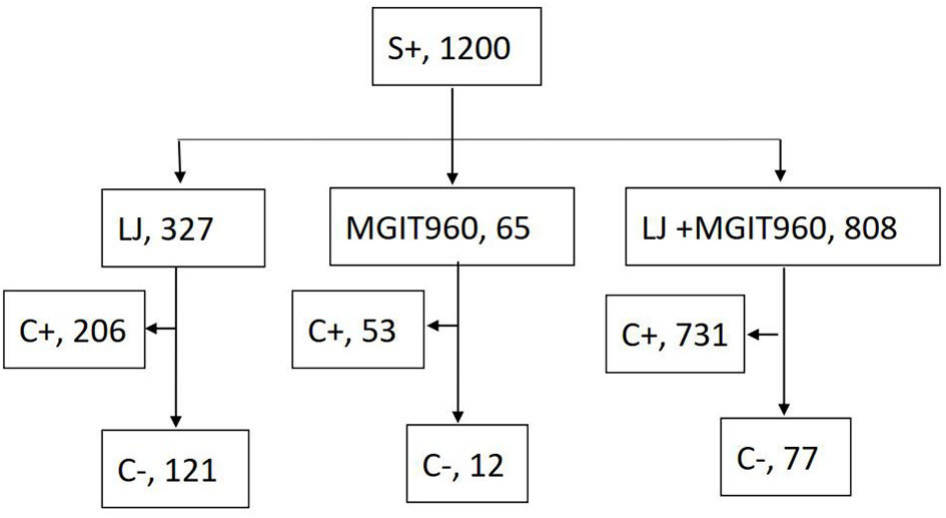
The simultaneous culture outcomes of the 1200 smear positive patients.

A total of 1,200 patients undertaking anti-TB treatments yielded S+ outcomes during the study period. In total, 990 (82.5%) had culture positive outcomes by any of the culture methods (Figure 1). Smear-positive, culture-negative patterns occurred in 210 of the 1,200 patients (17.5%). In total, 182 (86.7%) of the 210 S+/C– patients had follow-up bacterial examinations and/or traceable medical records. Among them, 100 were new cases, and 82 were retreated cases. The interval from the start of treatment to the current S+/C– outcome was <3 months for 72 (72.0%) patients and >3 months for 28 (28.0%) patients among new cases, <3 months for 24 (29.3%) patients, and >3 months for 58 (70.1%) patients among retreated cases. Retreated cases had the potential to obtain S+/C– outcomes later than the new cases during treatment (χ^2^ = 33.01, *p* < 0.001).

### 3.2. S+/C– outcome occurred in patients performed different culture types

In the group that performed LJ medium culture only, S+/C– outcome was observed in 121 of the 327 patients (37.0%); in the group that performed BACTEC MGIT960 medium culture only, S+/C–occurred in 12 of the 65 patients (18.5%); in the group performed both LJ and BACTEC MGIT960 medium culture, the S+/C–was observed in 77 of the 808 patients (9.5%), but 147 patients had MGIT960+/LJ– outcomes, while only 11 patients had LJ +/ MGIT960– outcomes. When solid and liquid cultures were independently considered, the S+/C– was observed more frequently among solid than liquid cultures (30.4%, 345/1135 vs. 11.5%, 100/873; *p* < 0.001, χ^2^ = 102.64).

### 3.3. The follow-up culture outcome after the occurrence of S+/C– outcome

Among the 182 patients with retractable records, 102 patients had been cultured at least once during the follow-up term and 35 (34.3%) patients had positive culture outcomes by any kind of culture method. Patients of all groups I, II, and III all had good chances to yield cultivatable bacilli during follow-up terms [45.5% (25/55), 25% (2/8), and 20.5% (8/39); Table 1]. More than half of them had positive outcomes within 1 month after the S+/C– outcome occurrence, while the leftover of them yielded the first positive culture during follow-up longer than 1 month. Additionally, 21 out of 51 retreated patients and 14 out of 51 new cases, who had follow-up cultures, cultivated live bacilli after S+/C– outcomes (41.2% vs. 27.5%). Retreated cases seemed to have more chances to present live bacilli during follow-up than the new cases after sporadic S+/C– outcomes were obtained during the treatment courses.

**Table 1:** The outcomes of the 102 patients with follow-up culture after the S+/C– evaluation enrollment.

**Group**	**Number**	**Culture positive No**.	**The time gap between S**+**/C– outcome and the first culture positive during follow up**		
			<**1 month**	**1–3 months**	>**3 months**
S+^*^/LJ–†	55	25	12	4	9
S+MGIT96–‡	8	2	0	2	0
S+/LJ–/MGIT960–	39	8	5	1	2

S+^*^, smear-positive; LJ–†, Lowenstein-Jensen (LJ) medium culture-negative; MGIT960–‡, BACTEC MGIT 960 liquid culture-negative.

### 3.4. The treatment response of patients who had no bacteriological evidence of live bacilli after the occurrence of S+/C– outcome

**Figure 2:**
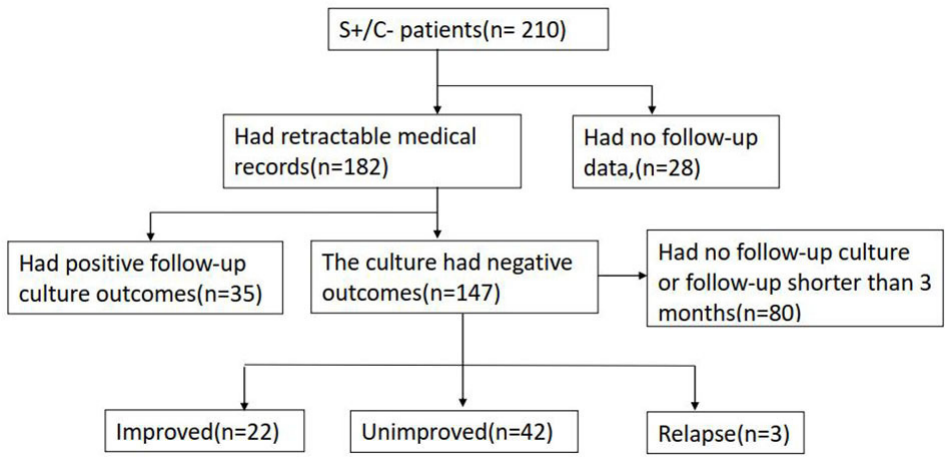
The status summary after S+/C– outcomes for the enrolled patients.

Among the 182 patients who had traceable medical records, 147 either had negative culture outcomes in the follow-up bacterial examinations or were not cultured afterward. In total, 80 out of these 147 patients were excluded because they had follow-up terms shorter than 3 months after obtaining the S+/C– outcome, which would be too short to evaluate the treatment response objectively. Therefore, 67 patients were eligible for treatment response evaluation by referring to the microbiological, radiological, and symptomatic data (Figure 2). The follow-up term ranged from 3 months to more than 2 years, and the average time was 13 months. Among them, 22 (32.8%, 22/67) were medically improved, and 3 (4.5%, 3/67) relapsed over the follow-up periods, whereas 42 (62.7%, 42/67) had unimproved conditions.

## 4. Discussion

A study in Tanzania observed 17.8% of their patients with newly diagnosed pulmonary tuberculosis produced S+/C– outcomes, and about 30% of these S+/C– patients were confirmed TB cases by repeated culture and/or molecular test (14). Since none of these patients had been exposed to any anti-TB drug when the sputa were collected, dead bacilli could not be a plausible explanation for the S+/C– outcomes. Other studies focused on S+/C– phenomena performed culturing using only LJ medium, and their observed S+/C– rates among patients who undertook treatment for a couple of months were between 20 and 32% (7, 8). It is well known that LJ medium is generally less sensitive than liquid culture and a study showed that S+/C– outcomes occurred less frequently with MGIT960 culture than with LJ medium (15). In this study, our data consistently demonstrated that S+/C–was more likely a result of lower sensitivity than of dead bacilli for LJ culture (1, 5, 16).

We obtained a little bit higher S+/C– rate (37.0%) for group I patients than other reposts, and we attribute this increase to the involvement of retreated patients in the study. All the other studies evaluated the S+/C– phenomenon among the small number of new cases, which may have different bacteriological conversion patterns than the retreated patients. Retreated patients often have more tough bacilli than new cases, for example, higher levels of drug resistance and long-term drug exposure may alleviate the vitality of the cells (17).

Totally, 102 patients performed culture at least once during the follow-up term and 35 (34.3%) patients had positive culture outcomes by any kind of culture method (Table 1). Although 67 patients did not present a positive culture outcome in the follow-up period, 24 of them had been cultured using an LJ medium only once. Therefore, it is reasonable to assume that some of those patients could produce positive outcomes if sufficient culturing had been performed. Nearly one-third of the patients harbored live bacilli after obtaining S+/C– outcome, which indicated that dead bacilli might not the reason for the previous S+/C– outcomes. Additionally, the higher sensitivity of the culturing, the less chance to yield live bacilli during follow-up terms. When neither LJ nor MGIT960 culture successfully cultivated bacteria in group III, dead bacilli might be a plausible explanation for the S+/C– outcomes. However, although lower than the other two groups, group III also acquired a 20.5% culture positive rate during follow-up periods. These results also negated dead bacilli as the major reason for S+/C– phenomenon. Furthermore, even dead bacilli in the sputa caused the S+/C– outcomes, according to our data, it did not suggest the absence of live bacteria in the lung of the patients at the time. Therefore, such patients should be treated as bacterial positive and may be subject to regimen adjustment.

Whether non-tuberculosis mycobacteria (NTM) was a cause of S+/C– outcome was evaluated. Species identification was performed on the recovered isolates after the S+/C– outcome of the enrolled patients, of which seven were identified as NTM. In total, seven patients were diagnosed with NTM infections, while five of them had positive cultures again after the S+/C– presented. Various studies have reported that NTM is a risk factor for S+/C– outcome; here, the proportion of NTM infection among the 210 cases was a little bit higher than the routine NTM isolation rate in the same laboratory (3.3 vs. 2.6%) (18), but the small number of patients does not make it conclusive evidence.

For the patient who either had negative culture outcomes in the follow-up bacterial examinations or no culture was performed afterward, the treatment response was followed up. According to our data, 67.2% (45/67) of the eligible cases had unfavorable prognoses within the follow-up term (including relapse and unimproved conditions). The prognostic evaluation outcomes did not support a good relationship between S+/C– and dead bacilli either.

According to our suggestion, a sporadic S+/C– outcome in clinical practice should first be taken as positive bacteriological evidence instead of assuming dead bacilli as a cause. However, since dead bacilli are an underlying reason for the S+/C– outcome, therefore, a comprehensive evaluation of clinical manifestations and chest radiographic findings and lateral bacteriological evidence are needed to interpret the outcomes appropriately. Some studies found that late smear positivity usually has no clinical significance and requires no specific action (17, 19), whereas Warring and Sutramongkole (20) recommended altering the drug therapy when multiple S+/C– specimens occur because the tuberculosis is becoming quiescent or inactive. Differences in the prevalence of TB and drug resistance in different settings can result in non-uniform conclusions. The differences in study designs can also lead to those different conclusions.

Our study has some limitations. As a national level referral hospital, a lot of patients only accepted periodical treatments here, which resulted in insufficient follow-up data for patient evaluation. This may lead to bias on less symptomatic and well-response patients, for whom the dead bacilli and S+/C– outcomes may be more related. Furthermore, the constitutions of the recruited patients (new case or retreated case; drug susceptible or resistant patients, time point when enrolled, and so on) may also cause bias in the conclusion and prompt further analysis to verify our inferential opinions.

## 5. Conclusion

In our studied set of patients, the sporadic smear positive and culture negative outcomes for sputa are more likely to be associated with low sensitivity of culture than with dead bacilli, especially when only LJ medium culture is used. In contrast to new cases, retreated cases easily produced S+/C– outcomes and had a higher change to cultivate live bacilli afterward. Despite our findings, further downstream work should be done to seek the underlying reason for S+/C– outcomes for patients with TB.

## Data availability statement

The raw data supporting the conclusions of this article will be made available by the authors, without undue reservation.

## Ethics statement

This study was approved by Medical Ethics Committee of Beijing Chest Hospital. The written consent was waived due to the retrospective design of the study.

## Author contributions

HH contributed to the study's conception and design. JL, XY, LD, FH, QL, YM, and YS were performed by material preparation, data collection, and analysis. HH and JL wrote the first draft of the manuscript. All authors commented on the previous versions of the manuscript, contributed to the article, and approved the submitted version.
